# Protective Roles of Interferon-Induced Protein with Tetratricopeptide Repeats 3 (IFIT3) in Dengue Virus Infection of Human Lung Epithelial Cells

**DOI:** 10.1371/journal.pone.0079518

**Published:** 2013-11-04

**Authors:** Yu-Lin Hsu, Shao-Fu Shi, Wan-Lin Wu, Ling-Jun Ho, Jenn-Haung Lai

**Affiliations:** 1 Graduate Institute of Medical Science, National Defense Medical Center, Taipei, Taiwan, R.O.C.; 2 Graduate Institute of Microbiology and Immunology, National Defense Medical Center, Taipei, Taiwan, R.O.C.; 3 Institute of Cellular and System Medicine, National Health Research Institute, Zhunan, Taiwan, R.O.C.; 4 Graduate Institute of Basic Medical Science, PhD Program of Aging, China Medical University, Taichung, Taiwan, R.O.C.; 5 Division of Allergy, Immunology, and Rheumatology, Department of Internal Medicine, Chang Gung Memorial Hospital, Chang Gung University, Tao-Yuan, Taiwan, R.O.C.; University of North Carolina at Greensboro, United States of America

## Abstract

Interferons (IFNs) are critical cytokines that regulate immune response against virus infections. Dengue virus (DV) infections are a major public health concern worldwide, and especially in Asia. In the present study, we investigated the effects and mechanisms of action of IFN-induced protein with tetratricopeptide repeats 3 (IFIT3) in human lung epithelial cells. The results demonstrated that DV infection induced expression of several IFITs, including IFIT1, IFIT2, IFIT3, and IFIT5 in A549 cells. Induction of IFIT3 by DV infection was also observed in human dendritic cells. In a knockdown study, we showed that a signal transducer and activator of transcription 2 (STAT2), but not STAT1 or STAT3, regulated DV-induced IFIT3 production. By using several different methods to evaluate cell death, we demonstrated that knockdown of IFIT3 led to cellular apoptosis. Furthermore, knockdown of IFIT3 induced the expression of several apoptotic regulators such as caspase 3, caspase 8, caspase 9, and Bcl-2-associated X protein (BAX). Such apoptotic effects and mechanisms were synergistically enhanced after DV infection. Moreover, under conditions of IFIT3 deficiency, viral production increased, suggesting an anti-viral effect of IFIT3. Interestingly, DV could suppress IFN-α-induced but not IFN-γ-induced IFIT3 expression, a phenomenon similar to the regulation of STATs by DV. In conclusion, this study revealed some mechanisms of IFIT3 induction, and also demonstrated the protective roles of IFIT3 following IFN-α production in DV infection of human lung epithelial cells.

## Introduction

 Dengue virus (DV) is a positive-strand RNA virus, and a member of the mosquito-borne *Flaviviridae* family of viruses. DV infections are a major public health concern worldwide, and especially in Asian countries. Two rare clinical manifestations, dengue hemorrhagic fever (DHF) and dengue shock syndrome (DSS), can cause fatal outcomes after DV infection. The annual occurrence of dengue fever (DF) is ~ 50-100 million cases worldwide, while ~ 250-500 thousand cases of DSS are reported annually [1]. Because the mechanisms of pathogenesis for DHF and DSS are largely unknown, effective therapies for these diseases are still lacking [2].

 Interferons (IFNs) are considered to be the most potent cellular cytokines for driving antimicrobial responses against intracellular virus infections [3,4]. It is estimated that 2,000 human and mouse IFN-stimulated genes (ISGs) have already been identified to date; however, most of these genes remain uncharacterized [5]. Currently, not much is known concerning how most of these ISG products function regarding their antiviral activities, target specificities, or mechanisms of action [6]. It is also difficult to link an IFN-induced protein to a specific antiviral effect because evidence suggests that several IFN-induced proteins may often act together to inhibit the same virus during different stages of its life cycle [6-8]. Furthermore, the presence of so many different *ISGs* is considered to allow for more potent antiviral activity, especially when a host encounters different families of viruses [3]. Systematic investigations into the specific anti-viral functions of different *ISGs* may offer greater insight into this issue [6,9]. 

 Among the products of various *ISGs*, the interferon produced by tetratricopeptide repeats genes (*Ifit*) has recently received a great deal of attention. Clustered in a locus on human chromosome 10, four members of this family of genes, namely *ifit1*, *ifit2*, *ifit3*, and *ifit5*, were evolutionarily conserved in mammals and amphibians [10]. A variety of stimuli can induce human and murine *ifit* genes through both IFN receptors and toll-like receptors: IFN-α/β are strong inducers, whereas IFN-γ is a weak inducer [10,11]. These IFIT proteins have unique helix-turn-helix structural motifs called tetratricopeptide repeats (TPRs) that are responsible for protein-protein and protein-RNA interactions [10-12]. The TPR motif is crucial for various cellular and viral functions such as protein transportation, translation initiation, cell migration, proliferation, antiviral signaling, and virus replication [10,13,14]. 

 We previously demonstrated that human dendritic cells (DCs), the most efficient antigen-presenting cells, can be infected by DV [15], and used microarray analysis to identify several interferon signaling-related genes induced in DV-infected DCs (data not shown). The induction of many of these identified genes has also been observed in the central nervous system of mice infected with DV Type-1 [16]. Human primary lung epithelial cells have been recognized as a primary target for DV infection, and the A549 cell line serves as a good host to study viral infection, especially in studies examining the effects of interferon [17-19]. In the present study, we focused on investigating the roles of IFIT3 in DV infection of A549 cells. The results revealed that IFIT3 induced after IFN stimulation might be critical for maintaining cell survival, and a deficiency of this molecule can result in increased apoptotic cell death, which is exaggerated in DV infection. We further demonstrated the crucial role of STAT2 in regulating DV-induced IFIT3 expression. Moreover, DV infection could downregulate the expression of IFIT3 induced by IFN-α, but not induced by IFN-γ. Collectively, our study contributes new insights for understanding the functions and roles of IFIT3, which is one of the *ISGs* whose expression is induced by DV infection in human lung epithelial cells.

## Materials and Methods

### Cell culture and reagents

Human lung epithelial cells A549 (Bioresource Collection and Research Center, Taiwan) were cultured in an F12 medium (Gibco-BRL, Life Technologies Corporation, Carlsbad, CA, USA) containing 10% fetal bovine serum (FBS, Gibco-BRL) in a humidified atmosphere containing 5% CO_2_ at 37°C. A variety of antibodies recognizing different molecules were purchased: IFIT3 and BAX (GeneTex Inc, Irvine, CA, USA); STAT1, STAT2, and STAT3 (Santa Cruz Biotechnology, Santa Cruz, CA, USA); phosphorylated STAT1, phosphorylated STAT2, phosphorylated STAT3, caspase 3, caspase 8, and caspase 9 (Cell Signaling, Beverly, MA, USA). The non-structural protein 3 (NS3) antibody was a gift from Dr. Chang-Chi Lin (National Defense Medical Center, Taiwan). 

### Establishment of Human DCs

DCs were generated from human peripheral blood mononuclear cells as previously described [20]. Monocytes were positively selected using a MACS cell isolation column following the manufacturer’s instructions (Miltenyi Biotech, Bergisch Gladbach, Germany) and cultured in a medium containing 800 U/mL granulocyte macrophage-colony stimulating factor and 500 U/mL interleukin-4 at a cell density of 1 × 10^6^ cells/mL. The culture medium was changed every other day and cells were used for experiments after 5–7 days of culture.

### DV preparation and infection of cells

DV was prepared as previously described [20]. Similar to many other studies [21,22], the mouse-adapted, neurovirulent prototype New Guinea C (NGC) strain of DV serotype 2 (DV2) was chosen as the viral strain for this study. In brief, DV2 NGCs were propagated in C6/36 mosquito cells in RPMI 1640 containing 5% heat-inactivated FBS and maintained at 28°C in a 5% CO_2_ atmosphere for 7 days. To prepare mock-infected supernatants, all of the procedures were identical, except that buffered saline was substituted for the virus inoculation. The virus titers in supernatants were determined by plaque-forming assays and stored at -70°C until use. Unless otherwise specified, A549 cells (1 × 10^5^/mL in culture medium) were infected with mock or DV at a multiplicity of infection (M.O.I.) = 5 for 2 h at 37°C. After absorbing the virus, the cells were washed, placed in fresh medium, and cultured for further analysis.

### RNAi silencing and short hairpin RNA (shRNA) knockdown

 For IFIT3 and STAT3 knockdown experiments, all siRNAs (Stealth RNAi™ siRNA, invitrogen, Life Technologies Corporation, Carlsbad, CA, USA) were transfected using RNAiMAX (Invitrogen) according to the manufacturer's instructions. The siRNA sequences used were listed as follows. IFIT3-1: GCAAUAUGCUAUGGACUAUUCGAAU; IFIT3-2: GCGCUACUGCAACCUUCAGAAAUAU; IFIT3-3: UGAGUUCCUGGAGACGGAAUGUUAU; STAT3-1: CAAUCAGGGAAGCAUCACAAUUGGC; STAT3-2: UGCUGUAGCUGAUUCCAUUGGGCCA. 

Cells were plated in antibiotic-free F12 medium. After reaching ~ 50% confluence, the cells were transfected with 10 nM siRNA. To determine the efficiency of protein knockdown, at 48 h post-transfection, cells were lysed in a RIPA buffer and immunoblotted with the indicated antibodies. Several specific shRNA constructs were designed and synthesized at the National RNAi Core Facility in Taiwan, ROC, and used to reduce STAT1 and STAT2 expression. One of the STAT1-specific shRNA constructs (clone ID: TRCN0000004265 with target sequence CCCTGAAGTATCTGTATCCAA) or STAT2-specific shRNA constructs (clone ID: TRCN0000364400 with target sequence TGTCTTCTGCTTCCGATATAA), or a control green fluorescence protein (GFP) shRNA construct (clone ID: TRCN0000072195 with target sequence GCGACGTAAACGGCCACAAGT) was co-transfected with the package and envelope plasmids into 293T cells to generate recombinant lentivirus carrying specific shRNA. The virus containing supernatants were harvested and the relative viral titers were determined by assaying the viability of A549 cells after puromycin selection according to a protocol from the National RNAi Core Facility. The viruses at M.O.I. = 1 were used to infect A549 cells. For infection, A549 cells were seeded one day before infection and then infected with a lentivirus carrying different shRNA constructs in the presence of 10 μg/mL Polybrene®. The cells were then cultured for an additional 10 days with regular replacement of puromycin (2 µg/mL) containing medium, and used for experiments.

### Quantitative RT/PCR (qRT-PCR)

 Total RNA from treated cells was isolated with TRIZOL® reagent (Invitrogen). RNA concentrations were measured using Nanodrop (ND 1000 V.3.1.0). Reverse transcription of purified RNA was performed using a random primer (Applied Biosystems, Life Technologies Corporation, Carlsbad, CA, USA). cDNA was used in quantitative real time PCRs, with the aid of a fluorescent Power SyBR® Green PCR master mix (Fermentas, Glen Burnie, MD, USA) and 7500 Real Time PCR System (Applied Biosystems). All values were normalized to the level of GAPDH mRNA. All assays were performed in triplicate and repeated in 3 independent experiments. The primers used were as follows: IFIT1, sense (5’-TCTCAGAGGAGCCTGGCTAA-3’), antisense (5’-TGACATCTCAATTGCTCCAG-3’); IFIT2, sense (5’-AAGAGTGCAGCTGCCTGAA-3’), antisense (5’-GGCATTTTAGTTGCCGTAGG-3’); IFIT3, sense (5’-GAACATGCTGACCAAGCAGA-3’), antisense (5’-CAGTTGTGTCCACCCTTCCT-3’); IFIT5, sense (5’-GGCCAAAATAAAGACGCCCT-3’), antisense (5’-GACCAGGCTTCGTACTTCTTC-3’); IFN-β, sense (5’-CGCCGCATTGACCATCTA-3’), antisense (5’-GACATTAGCCAGGAGGTTCT-3’).

### MTT assay

 The MTT [3-(4, 5-dimethylthiazol-2-yl)-2, 5- diphenyl-tetrazolium bromide] colorimetric assay was used to determine cell survival rates. Treated cells were incubated with MTT (100 µL/well), (USB Corporation, MA, USA) at 37°C in a humidified 5% CO_2_ atmosphere overnight. Then, the MTT solution was removed and the formazan crystals were dissolved in 100 μL/well DMSO (Sigma, St. Louis, MO, USA) and incubated for 30 min at 37°C. The absorbance at 540 nm was measured using a microplate reader (Sunrise, TECAN, Männedorf, Switzerland) and 100% viability was defined as the absorbance of the control. Cell survival rates were calculated according to the following equation: survival rate = [experimental absorbance value / control absorbance value] × 100%.

### SubG1 assay

 A549 cells were collected and washed with PBS, and then fixed in iced alcohol overnight at -20°C. After washing twice with PBS, the cells were stained with 50 μg/mL propidium iodide (PI, Sigma) containing 200 μg/mL RNase A (Sigma) in PBS at room temperature for 30 min. After staining with PI, quantification of the SubG1 population was carried out using a flow cytometer (BD Biosciences, CA, USA). The calculation of synergistic effects was listed as follows. The effect of DV infection = (DV/si-Ctl – mock/si-Ctl), the effect of IFIT3 knockdown = (mock/siIFIT3 – mock/si-Ctl) and the effect of DV infection and IFIT3 knockdown = (DV/siIFIT3 – mock/si-Ctl). Ctl stands for control.

### Caspase 3 activity measurement

The caspase 3 activity assay was performed according to the manufacturer's protocol (PE Active Caspase-3 Apoptosis Kit, BD Pharmingen™). A549 cells were collected and washed twice with cold PBS, then suspended in Cytofix/Cytoperm™ solution and incubated for 20 min on ice. After washing twice with Perm/Wash™ buffer, the cells were incubated with specific antibody for 30 min at room temperature and then were analyzed by flow cytometry.

### Annexin-V and 7-amino-actinomycin D (7-AAD) staining assay

The Annexin-V and 7-AAD staining assay was performed according to the manufacturer's protocol (PE Annexin V Apoptosis Detection Kit I, BD Pharmingen™). The cells were washed twice with cold PBS and then PE (phycoerythrin) Annexin V and 7-AAD was added into binding buffer. The reaction proceeded for 15 min at room temperature in the dark. The cells were then analyzed and quantified using flow cytometry.

### Terminal deoxynucleotidyltransferase-mediated dUTP-biotin nick end labeling (TUNEL) assay

 The TUNEL assay was performed according to the manufacturer's protocols (*In Situ* Cell Death Detection Kit, Roche Diagnostics Corp., IN, USA). Cells were fixed in 4% paraformaldehyde for 1 h at room temperature, and then permeabilized with 0.1% sodium citrate and 0.1% Triton X-100 (Sigma Aldrich) in ice for 2 min. After washing with PBS, cells were incubated with the terminal deoxynucleotidyl transferase (TdT) buffer containing fluorescein labeled dUTP for 1 h at 37°C in the dark. TUNEL-positive cells were analyzed and quantified using a FACS flow cytometer. Alternatively, after cytospinning, the numbers of TUNEL positive cells in 3 random fields were counted under a fluorescence microscope. 

### Western blotting

 ECL western blotting (Amersham, GE Healthcare Life Science, Uppsala, Sweden) was performed as previously described [20]. In brief, the cells were pelleted, washed several times, and resuspended in a lysis buffer. The mixture was then vortex mixed, centrifuged, and the supernatant was collected. Protein concentrations were determined with protein assay dye reagent (Bio-Rad, Bio-Rad Laboratories, Hercules, CA, USA). Equal amounts of proteins obtained from whole cellular extracts were analyzed on a 10% SDS PAGE gel and transferred to a nitrocellulose filter. For immunoblotting, the nitrocellulose filter was incubated with TBS-T containing 5% nonfat milk or 5% BSA (for p-STAT2, p-STAT3, and total STAT3 antibodies) for 1 h, and then blotted with antisera against individual proteins overnight at 4°C. The filter was then washed 3 times with TBS-T and incubated with a secondary antibody at a concentration of 1/1000 - 1/5000 for 1 h. The filter was then incubated with the substrate and exposed to x-ray film. 

### NS3 protein staining and plaque assay

 For determination of viral NS3 protein expression, cells were first permeabilized with 0.5% saponin (Sigma). After incubation for another 30 min, the anti-DV NS3 antibodies were added. After washing, goat anti-mouse antibodies conjugated with fluorescein isothiocyanate were added and incubated for another 30 min. Finally, the samples were analyzed by flow cytometry. Various dilutions of virus were added to 80% confluent baby hamster kidney (BHK-21) cells and incubated at 37°C for 2 h. After adsorption, the cells were washed and overlaid with 3 mL of RPMI 1640 containing 1% low-melting-temperature agarose (SeaPlaque; FMC BioProducts, Philadelphia, PA, USA), 1% penicillin, 1% streptomycin and 2% FBS. The cells were incubated for 7 days, and then fixed with 2% formaldehyde and stained with 0.5% crystal violet. The numbers of plaques were counted and results were recorded as plaque forming units per milliliter.

### Immunocytochemistry

 Cells were plated on chamber slides which were then soaked with 75% EtOH and exposed to UV radiation. After DV infection for 24 h, the cells were fixed with 4% paraformaldehyde for 20 min at room temperature. After permeabilization with 1% Triton X-100 for 20 min, the cells were blocked with PBS containing 1% BSA and 0.1% Triton X-100 for 1 h, and then incubated sequentially for 2 h with anti-IFIT3 antibodies (rabbit monoclonal anti-human; GeneTex), followed by 1 h with a secondary antibody (Goat anti-rabbit IgG-FITC, at a 1:25 dilution; Abcam, Cambridge, ENG) at room temperature. Slides were washed with PBS at each step and the nuclei were stained with DAPI (4′,6-diamidino-2-phenylindole; Sigma) for 10 min before microscopic analysis. 

### Construction of pCR3.1-IFIT3-Flag and over-expression in A549 cells

 pCR3.1-IFIT3-Flag was constructed by inserting PCR-amplified full-length IFIT3 coding sequences created at both NheI and XhoI enzyme cutting sites into the pCR3.1-Flag vector. Successful ligations were conﬁrmed by sequencing. pCR3.1-IFIT3-Flag or a control plasmid (pCR3.1) was transfected into A549 cells (1 × 10^5^/mL) by use of Fugene HD (Roche, Penzberg, Upper Bavaria, Germany). DNA plasmids were mixed with the provided reagent at a ratio of 2:3 in Opti-MEM (Invitrogen). After incubation for 15 min at room temperature for complex formation, the mixture was added drop-wise to the cells. The IFIT3 cloning primers used were: sense (5’- gacacgctagcatgagtgaggtcacc-3’); antisense (5’-caataactcgaggttcagttgctctgagt-3’).

### Statistical analysis

 The results were expressed as the mean ± SD of triplicate experiments. Statistical comparisons were performed using the student’s T test or one-way analysis of variance (ANOVA). When ANOVA showed significant differences between groups, Bonferroni’s post-hoc test was used to determine the specific pairs of groups between which statistically significant differences occurred. A P value < 0.05 was considered statistically significant. The symbol “*” indicates values that are significantly different from the control (*P < 0.05; **P < 0.01; ***P < 0.001).

## Results

### DV infection induced IFIT3

**Figure 1:**
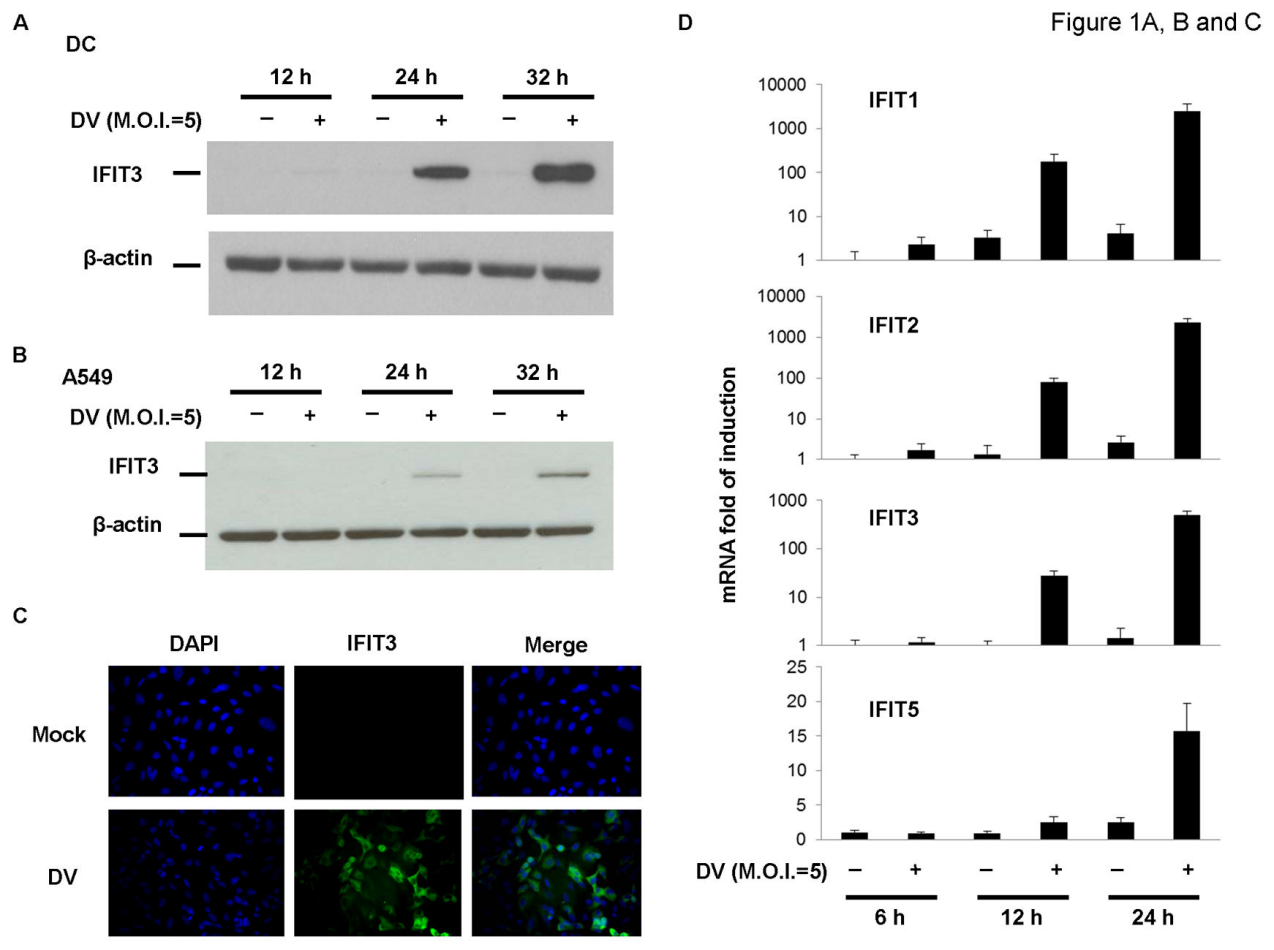
DV infection induced IFIT expression. Human DCs (A) or A549 cells (B, C, and D) at 1 х 10^6^ or 1 х 10^5^ cells/mL were infected by mock or DV at various time points. Total cell lysates were collected and the expression of IFIT3 or β-actin was determined by western blotting (A and B) or immunocytochemical staining (C). Expression of mRNAs of *ifit1*, *ifit2*, *ifit3*, and *ifit5* genes was determined by quantitative RT-PCR (D). The data shown are from 3 independent experiments.

A microarray analysis examining genes that were up- or downregulated in DV-infected DCs, showed the induction of several IFN and IFN signaling-related genes (data not shown). One of those genes, IFIT3, was further characterized, because the function of IFIT3 is less understood. Consistent with results of microarray analysis, protein levels of IFIT3 increased after DV infection of human DCs (Figure 1A). Similar findings were demonstrated in the DV-infected human lung epithelial cell line (A549 cells), (Figure 1B). This finding was further supported by results of immunocytochemical staining (Figure 1C). The induction of IFIT3 by DV infection is believed to be mediated through secreted IFN-α, because the effect was successfully blocked by neutralizing monoclonal antibodies recognizing IFN receptors (Figure S1). DV infection also induced expression of mRNAs by several *ifit* genes, including *ifit1*, *ifit2*, and *ifit5*, although the intensities and kinetics varied (Figure 1D). 

### Induction of IFIT3 Is STAT-2-dependent

**Figure 2:**
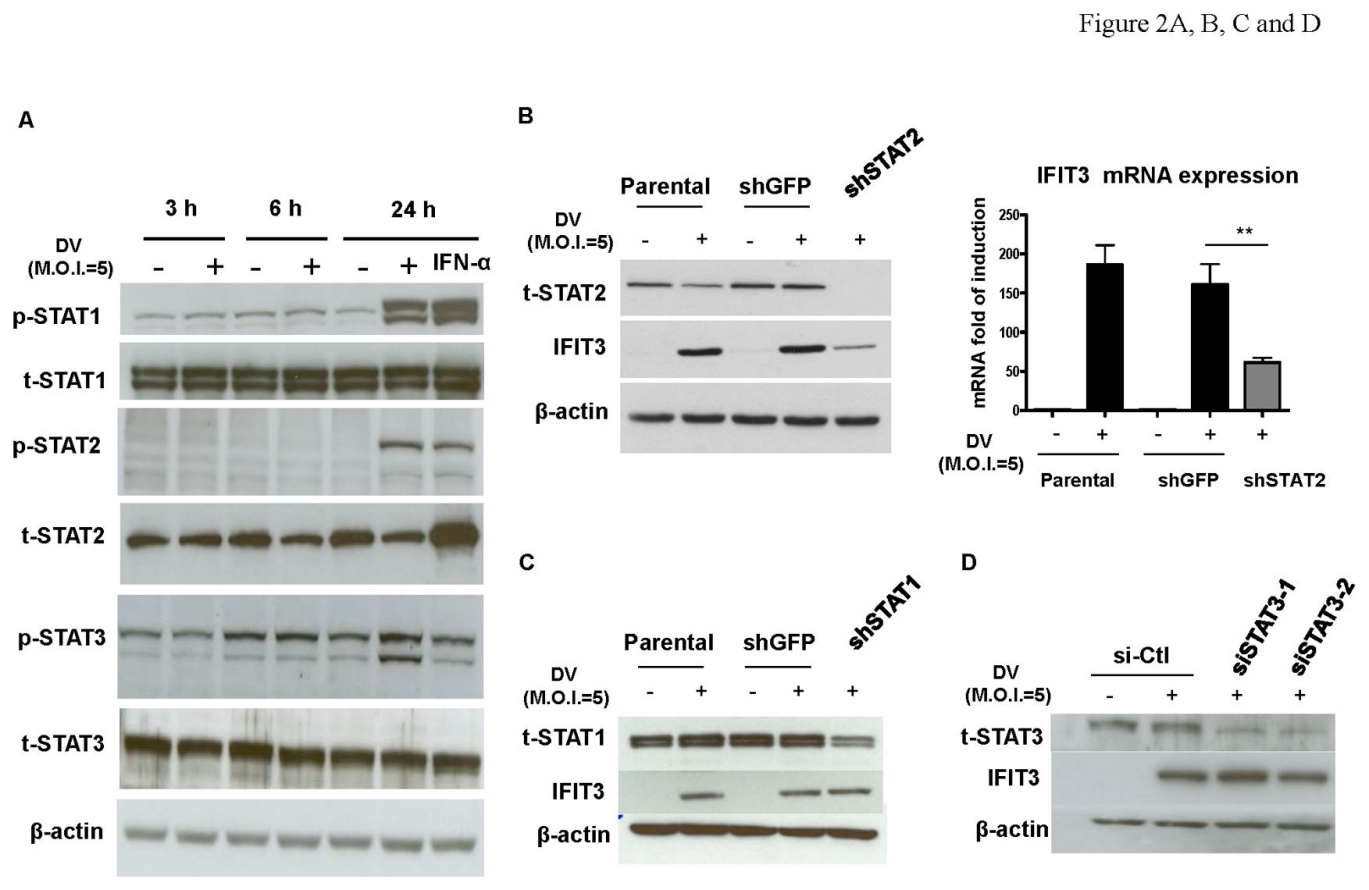
Induction of IFIT3 is STAT-2-dependent. A549 cells were infected by mock or DV for 3, 6, and 24 h and protein levels of both phosphorylated and non-phosphorylated STAT1, STAT2, and STAT3 were analyzed by western blotting (A). Treatment with 1000 units IFN-α was used as a positive control. Expression of IFIT3 in DV-infected A549 cells with knockdown of either STAT2 (B), STAT1 (C) or STAT3 (D) was determined by western blotting (B, C, and D) or quantitative RT/PCR (B). Both shRNA and siRNA were used as the approaches for STAT1/STAT2 and STAT3, respectively, as described in Materials and Methods. Knockdown with shGFP or si-Ctl was used as a negative control. Data show representative results and analysis pooled from at least 3 independent experiments. The analysis was performed by ANOVA as described in Materials and Methods. **P < 0.01. Ctl stands for control.

The potential involvement of STAT transcriptional factors [10] in DV-induced IFIT3 expression was investigated. A549 cells were infected by mock or DV at various time points, and the protein levels of both phosphorylated and non-phosphorylated STAT1, STAT2, and STAT3 were analyzed. Similar to observations in DCs [20], DV infection induced expression of phosphorylated STAT1, STAT2, and STAT3 in A549 cells (Figure 2A). Interestingly, in contrast to STAT1 and STAT3, DV infection attenuated total STAT2 levels; however, the mechanism of this attenuation is unclear. To determine whether induction of STAT proteins may play a role in regulating expression of IFIT3, both shRNA and siRNA were used to knockdown the protein levels of STAT1, STAT2 or STAT3 as described in Materials and Methods. Results revealed that knockdown of STAT2 successfully reduced both IFIT3 mRNA and IFIT3 protein levels (Figure 2B). In contrast, knockdown of STAT1 or STAT3 did not affect DV-induced IFIT3 expression (Figures 2C and 2D). The statistical analysis for the band intensity of western blotting in Figure 2 (A to D) was shown in the Figure S2 (A to D).

### Knockdown of IFIT3 enhanced DV production

**Figure 3:**
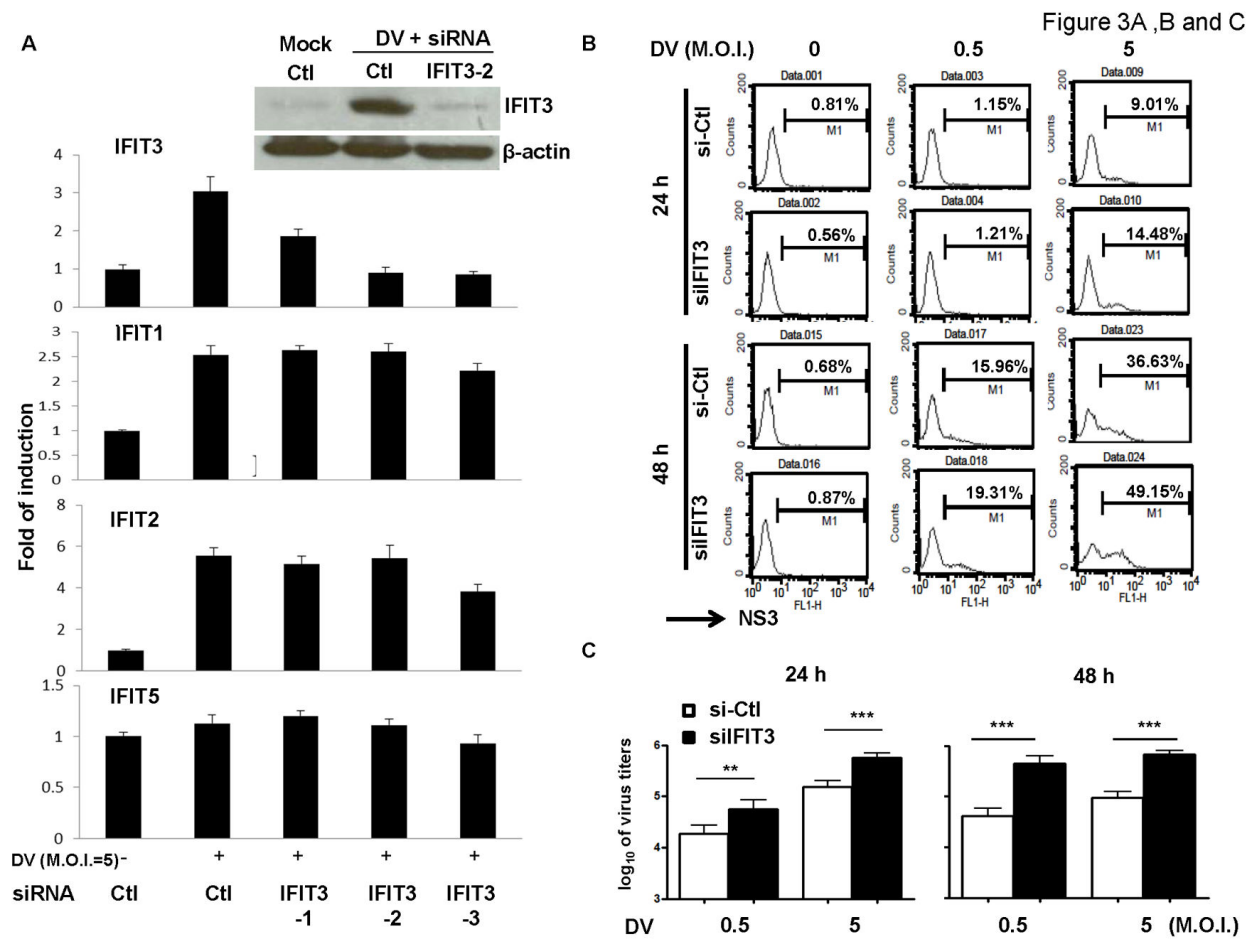
IFIT3 knockdown with siRNA without affecting other *ifit* genes increased DV production. A549 cells were transfected with different siRNAs (si-Ctl, siIFIT3-1, siIFIT3-2 or siIFIT3-3) for 24 h and then infected with mock or DV for 13 h. Expression of mRNA of *ifit* genes and IFIT3 protein was determined by quantitative RT/PCR and western blotting, respectively (A). Data show results of 3 independent experiments. A549 cells transfected with control siRNA (si-Ctl) or IFIT3 siRNA (siIFIT3-2) for 24 h were infected by mock or DV at M.O.I. = 0.5 or 5 for an additional 24 or 48 h. Cells were collected for measurement of expression of intracellular NS3 by flow cytometry (B). Supernatants were collected to determine virus titers by plaque assays (C). Data show results pooled from at least 3 independent experiments. The analysis was performed by ANOVA as described in Materials and Methods. **P < 0.01, ***P < 0.001. Ctl stands for control.

We next synthesized 3 small interference RNAs (siRNAs) to knockdown the expression of IFIT3. The results suggested that neither siIFIT3-1 nor siIFIT3-2 affect expression of other *ifit* genes (Figure 3A). Therefore, siIFIT3-2 was chosen as an interference tool for all subsequent studies. A549 cells transfected with control siRNA (si-Ctl) or siIFIT3 were infected with mock or DV at M.O.I. = 0.5 or 5 for an additional 24 or 48 h, and then collected for measuring expression of intracellular NS3 by flow cytometry. The results demonstrated that DV infectivity increased in cells deficient in IFIT3 (Figure 3B). Also, an increase in viral production was observed in cells deficient in IFIT3 compared to those transduced with the siRNA control (Figure 3C). 

### IFIT3 knockdown synergistically enhanced cell death in DV-infected A549 cells

**Figure 4:**
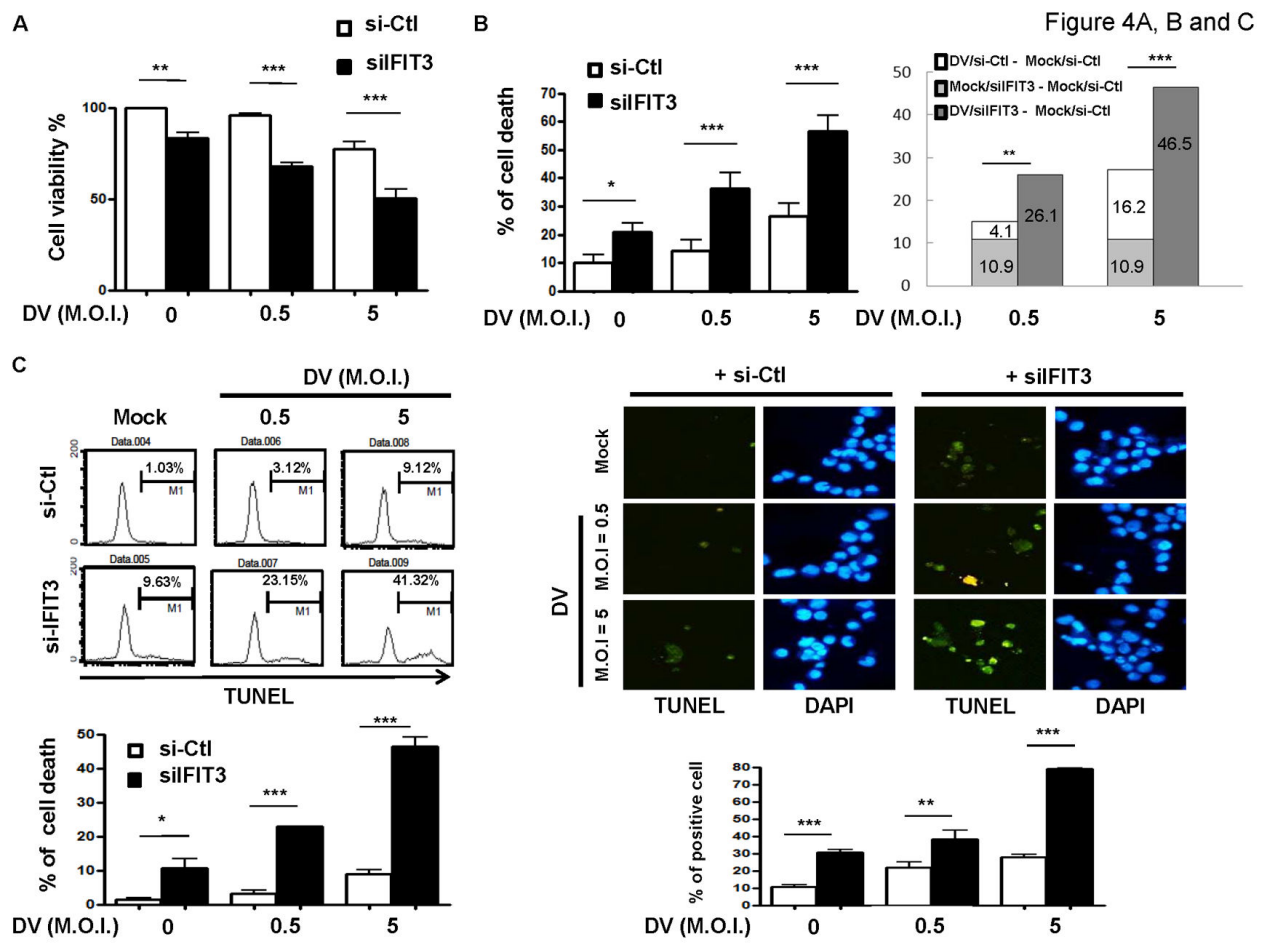
Synergistic pro-apoptotic effects of both knockdown of IFIT3 and DV infection. A549 cells transfected with control siRNA (si-Ctl) or IFIT3 siRNA (siIFIT3) for 24 h were infected with mock or DV at M.O.I. = 0.5 or 5 for an additional 48 h. Cell viability was determined by MTT assays. Mock-infected cells treated with control siRNA transfection were taken as 100%, and OD values from individual conditions were normalized by the value of the control (% of control; A). DNA content was determined by sub-G1 analysis (left panel in B), and the synergistic effects were calculated (right panel in B). Determination and visualization of cell apoptosis were also performed by TUNEL assays using flow cytometry and immunofluorescent staining (C). Data show representative results and analysis pooled from at least 3 independent experiments. The analysis was performed by ANOVA as described in Materials and Methods. *P < 0.05, **P < 0.01, ***P < 0.001. Ctl stands for control.

Because DV infection can potentially cause cell death [23] and IFIT2 has been shown to be protective against vesicular stomatitis virus infection-induced death in mice [24], the potential protective roles of IFIT3 in DV-induced death of A549 cells were examined. To determine the effects of IFIT3 knockdown on DV-induced cell death, mock- or DV-infected A549 cells with a deficiency of IFIT3 were analyzed using different approaches. MTT assays demonstrated that knockdown of IFIT3 caused significantly more cell death in both mock-infected and DV-infected A549 cells (Figure 4A). These results were confirmed by measuring the number of cells in sub-G1 phase. Accordingly, the percentages of cell death were 4.1% (M.O.I. = 0.5) and 16.2% (M.O.I. = 5). Knockdown of IFIT3 caused 10.9% cell death. A combination of both DV infection and IFIT3 knockdown resulted in increased cell death up to 26.1% (M.O.I. = 0.5) and 46.5% (M.O.I. = 5), which were much higher than individual conditions (Figure 4B). The numerical analysis revealed that a reduction of IFIT3 plus DV infection worked synergistically to induce a significant increase in cell death. The death of cells appeared to involve an apoptotic effect, because TUNEL analysis using both flow cytometry and immunofluorescent staining revealed synergistically increased percentages of death among cells exposed to both DV infection and IFIT3 deficiency (Figure 4C). In considering that it is usually hard for viruses to propagate in dying cells, the correlation of kinetics between viral production and cell death under the condition with or without IFIT3 knockdown was further analyzed. As shown in the lower panel of Figure S3A, viral production peaked as early as 24 h after infection. In contrast, compared to mock infection, virus infection-induced cell death became prominent 48 h after infection (Figure S3A upper panel). The results also showed that the effect of virus infection-induced cell death appeared 48-72 h after virus infection in cells with knockdown of IFIT3 (Figure S3B). Meanwhile, we observed that DV-induced production of IFN-β decreased although without statistical significance in cells with deficiency of IFIT3 (Figure S3C). These observations might explain in part why knockdown of IFIT3 that caused cell death also resulted in increased viral production.

### Induction of caspases in DV infection and effects with IFIT3-knockdown

**Figure 5:**
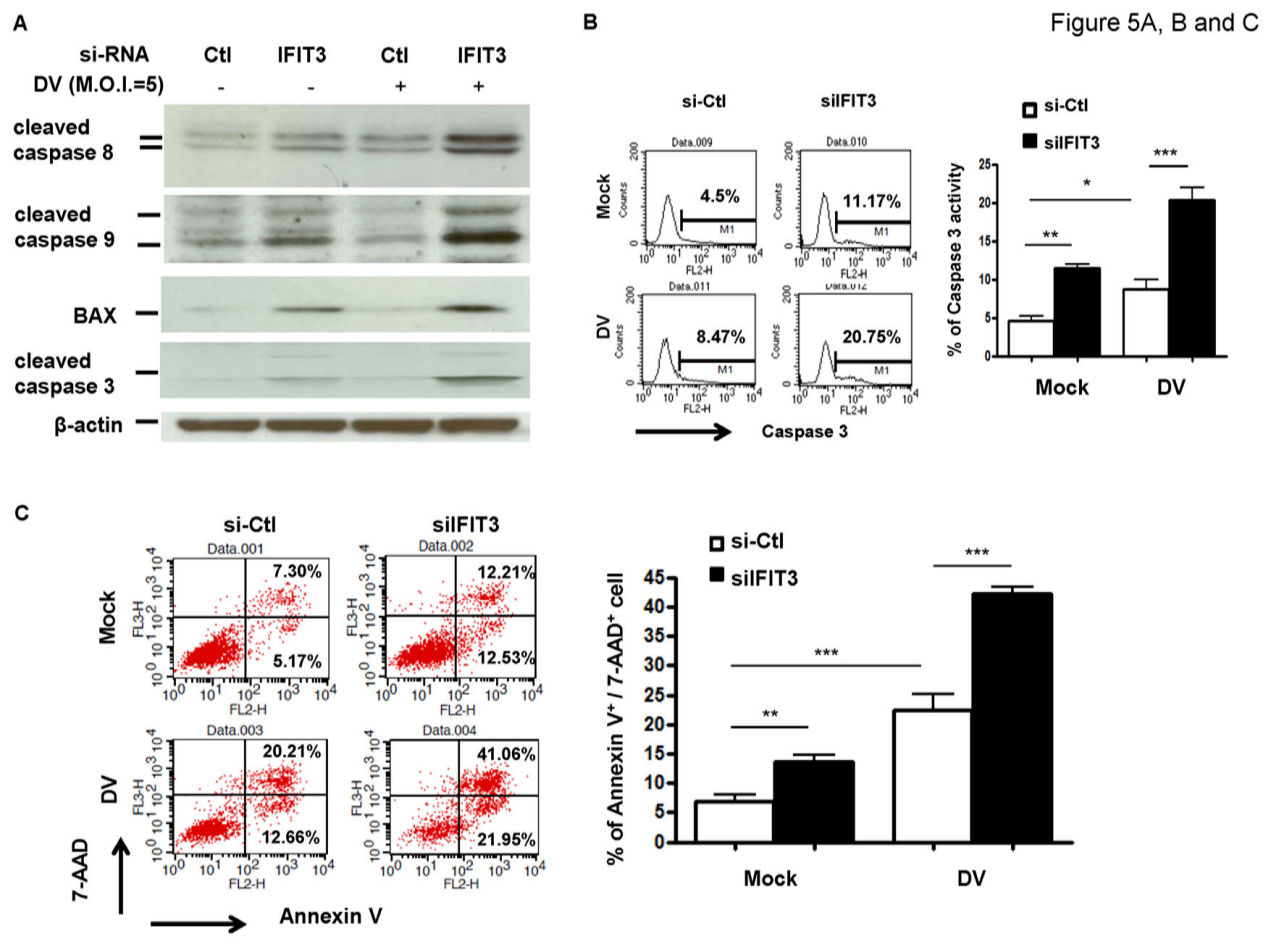
Induction of apoptotic molecules by deficiency of IFIT3. A549 cells transfected with control siRNA (si-Ctl) or IFIT3 siRNA (siIFIT3) for 24 h were infected by mock or DV at M.O.I. = 5 for another 24 h. The cleaved proteins, including caspase 8, caspase 9, caspase 3 and BAX were determined by western blotting (A). Caspase 3 activity (B) or Annexin V and 7-AAD (C) were determined by flow cytometry analysis at postinfection 48 h. The representative results and the analysis pooled from at least three independent experiments were shown. The analysis was performed by ANOVA as described in Materials and Methods. *P<0.05, **P<0.01, ***P<0.001. Ctl stands for control.

To further confirm the pro-apoptotic effects of IFIT3 deficiency, the expression of several pro-apoptotic molecules was determined. The cell lysates collected from IFIT3-knockdown cells infected with DV or mock were analyzed by western blotting to determine the expression of cleaved caspase 3, caspase 8, and caspase 9, as well as BAX (Figure 5A). The statistical analysis on band intensity was determined and shown in Figure S4 (A and B). The IFIT3-knockdown cells infected by DV or mock were also analyzed to determine the caspase 3 activity (Figure 5B) and annexin V and 7-AAD staining (Figure 5C), different approaches determining cell death, by flow cytometry. Although knockdown of IFIT3 by itself could induce expression of these pro-apoptotic molecules related to cell death, the results clearly showed that IFIT3-knockdown significantly enhanced the apoptotic effects of DV infection.

### Overexpression of IFIT3 enhanced cell survival

**Figure 6:**
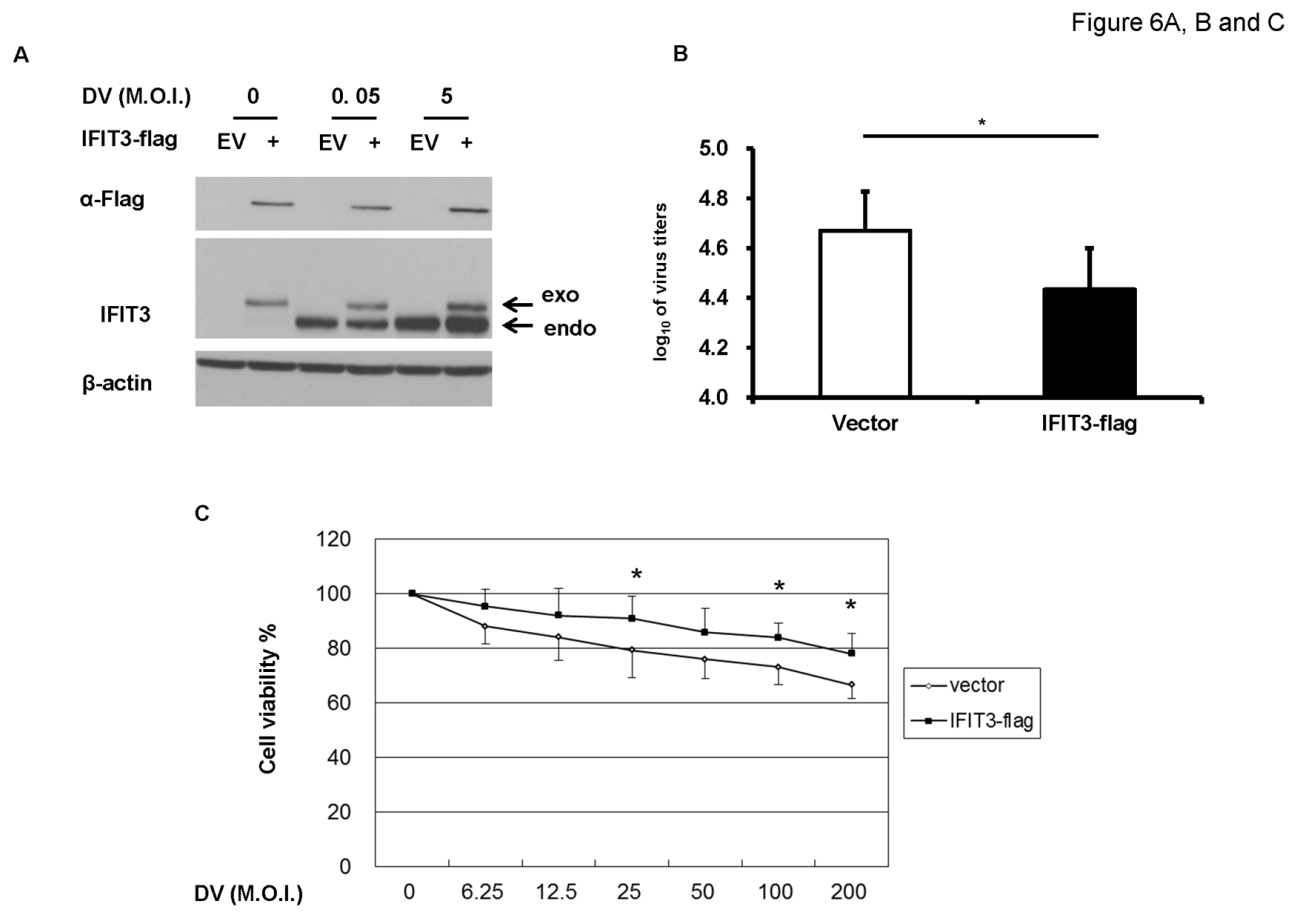
Overexpression of IFIT3 significantly enhanced cell survival and blocked DV replication. A549 transfected with IFIT3-flag or empty vector (EV) for 24 h were infected by mock or DV at M.O.I. = 0.05 or 5 for 48 h. The expression of endogenous and exogenous IFIT3 was determined by western blotting as described in the Materials and Methods (A). The supernatants were collected for determining virus titers by plaque assays (B). After transfection, the cell were reseeded onto 96 well plate overnight and then infected by mock or DV at various M.O.I. = 6.25 to 200 for 48 h, the cell viability was determined by MTT assay (C). The representative results and the analysis pooled from at least three independent experiments are shown. The analysis was performed by student’s T test (B) or ANOVA (C) as described in Materials and Methods. *P<0.05.

Because a deficiency of IFIT3 increased cell death, we examined whether overexpression of IFIT3 might have a rescue effect. The results described in Figure 6A show that compared to mock infection, DV infection at M.O.I. = 0.05 slightly induce expression of IFIT3; thus the transfection with plasmid encoding IFIT3-flag modestly increased the amount of cellular IFIT3. These results demonstrate that the modestly increased levels of IFIT3 significantly reduced virus titers (Figure 6B) and increased cell survival (Figure 6C) in DV-infected cells. Due to technical limitation of low transfection efficiency in delivering IFIT3-flag into A549 (lower than 20%), the effect of overexpression of IFIT3-flag on viral production was not promising although the statistical analysis was still significant. Another reason to explain this observation is that IFIT3 might need to cooperate with IFIT1 and IFIT2 to effectively block viral propagation; therefore, the modestly increased expression of IFIT3 could only achieve limited anti-viral effects. These collective results appear to indicate that a basal level of IFIT3 is required to inhibit viral production; however, the presence of higher levels of IFIT3 may exert stronger anti-viral effects in DV infected A549 cells.

### DV infection blocked IFN-α but not IFN-γ induced IFIT3 induction

**Figure 7:**
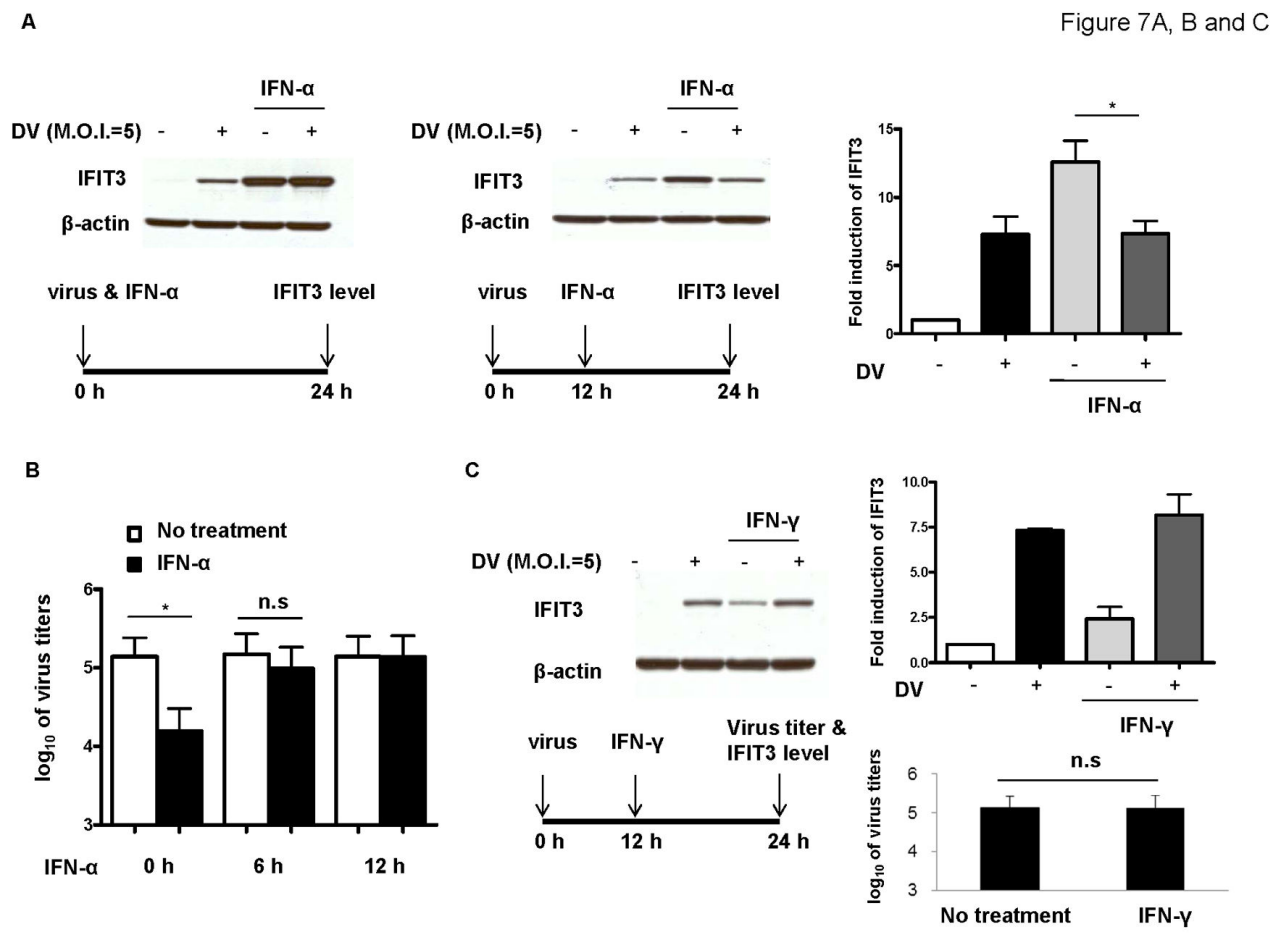
Effects of simultaneous or post-infection treatment with IFN-α or IFN-γ on IFIT3 expression and DV production. Shortly after viral absorption, DV-infected A549 cells (1 x 10^5^/mL) were treated with 1000 units IFN-α and incubated for an additional 24 h. Alternatively, 1000 units IFN-α were added into the culture medium 12 h after virus infection and incubated for an additional 12 h. Expression of IFIT3 was determined by western blotting, and relative band intensities were quantified, right panel (A). IFN-α was added simultaneously with DV infection or 6 h or 12 h after DV infection. After incubation for additional 12 h, the supernatants were collected and virus titers were measured by plaque assays (B). Similar to (A), A549 cells infected by mock or DV for 12 h were treated with 100 units IFN-γ, and the cell cultures were then maintained for an additional 12 h. IFIT3 levels in cell lysates and virus titers in supernatants were determined (C). Data represent results from 3 independent experiments. The analysis was performed by ANOVA (A and B) or student’s T test (C) as described in Materials and Methods. *P < 0.05. n.s: no significance.

We previously demonstrated that DV infection could evade INF-α but not IFN-γ anti-viral effects through regulating STAT expression [20]. We then investigated whether IFIT3 could also serve as another target for DV to manipulate anti-viral activities of IFNs. A549 cells were infected by DV and treated with IFN-α at the same time or infected by DV for 12 h and then followed by IFN-α treatment as the figure showed (Figure 7A). The expression of IFIT3 by these cells was determined. The results showed that when DV infection occurred 12 h before treatment with IFN-α, IFN-α-induced IFIT3 expression was significantly suppressed (Figure 7A, middle and right panels). Within 6 h of infection, DV already gained capacity to downregulate although non-significantly IFN-α-induced IFIT3 expression (Figure 7A left panel and Figure S5). Plaque assays conducted to determine virus titers showed that if IFN-α was added 12 h after DV infection, then it completely lost its anti-viral activity (Figure 7B). In contrast, IFN-γ-induced IFIT3 expression and anti-viral activity were not affected by DV infection (Figure 7C). We then examined the significance of DV-downregulated IFIT3 expression induced by IFN-α stimulation in A549 cells. Cells transfected with control siRNA or IFIT3 siRNA for 24 h were pretreated with IFN-α for 5 h and then infected by mock or DV for another 24 or 48 h. The supernatants and cells were collected for determining virus titers by plaque assays (Figure S6A) and for measuring expression of intracellular NS3, a reflection of viral replication, by flow cytometry (Figure S6B), respectively. We were surprised to observe that compared to the control, knockdown of IFIT3 did not affect IFN-α-mediated suppression of viral production (Figure S6A and 6B). These results seem to indicate that although DV could downregulate IFN-α-induced IFIT3 expression, such an effect did not help protect virus against IFN-α-mediated anti-viral replication. Thus, the significance of downregulation of IFN-α-induced IFIT3 expression by DV is currently unclear. A mechanical model was proposed to explain the regulation of IFIT3 in DV-infected A549 cells (Figure S7).

## Discussion

IFIT3 was first identified as an all-trans-retinoic acid- and IFN-induced gene in acute promyelocytic leukemia cells [25]. The fact that induction of IFIT3 in response to IFN-α stimulation has been described in a variety of tissue cell lines, suggests crucial roles for this protein in IFN-α-mediated actions [26]. Also, the analogues of IFIT3 have been identified in many different species [27]. The diffuse distribution of IFIT3 in cytosol further indicates its important role in various cellular activities [25]. The induced expression of IFIT3 by DV infection was demonstrated by both western blotting and immunocytochemical staining. In addition, consistent with the results of microarray analysis in DV-infected DCs, mRNAs of *ifit1*, *ifit2*, *ifit3*, and *ifit5* genes were induced by DV infection of A549 cells. Although not examined in detail, it is likely that much like IFIT3, the production of IFIT1, IFIT2, and IFIT5 in DV-infected A549 cells might also be regulated in an autostimulating manner, and be dependent on the production of IFN-α. Results showing that IFIT1, IFIT2, and IFIT3, but not IFIT5, were induced with similar kinetics by DV infection suggest the possibility of co-regulation, and also the coordination and non-redundant functioning of these molecules in IFN-mediated signaling events, as observed in neurons [25]. 

IFN stimulation may cause many diverse events, including apoptotic cell death [28]. The knockdown studies suggest that IFIT2 may be at least one of the possible factors responsible for IFN-α-stimulated apoptotic death [29]. IFIT2 can associate together with IFIT1 and IFIT3 and mediate cellular apoptosis; however, binding of IFIT3 negatively regulates the apoptotic effects of IFIT2 [29]. It is thus suggested that induction of IFIT3 by IFN-α is a primary regulator of IFIT2-induced cell death. Nevertheless, in our study, the role of IFIT3 appeared to be more complicated. Knockdown of IFIT3 by itself, in the absence of influencing IFIT2 or IFIT1 could cause cell death, and this effect was further enhanced in the presence of DV infection. Several apoptosis-related molecules such as caspase 3, caspase 8, caspase 9, and BAX appeared to be induced under conditions of reduced IFIT3, and the expression of these molecules was further enhanced by DV infection. It is evident that along with secretion of IFN-α, production of IFIT3 might be responsible for some portion of cell survival mechanisms, and reduction of IFIT3 may result in cellular apoptosis. Overexpression of the *ifit3* gene increased cell survival, suggesting that this molecule functions as a house-keeping molecule for IFN-mediated survival effects. Interestingly, the ectopic expression of IFIT3 in U937 cells results in an increased accumulation of cells in G(1)/S transition and growth arrest [26]. As reported by Fensterl et al [24], in the absence of IFIT2, the vesicular stomatitis virus replicates and very efficiently produces viral progenies in the brains of mice; however, the anti-viral effect of IFIT2 was not observed in other organs such as the lungs and liver. Furthermore, such a protective effect of IFIT2 was not demonstrated in neurotropic RNA virus infections. These results may be due to the tissue-, virus-, and ISG-specific characteristics of antiviral actions of downstream IFN effector molecules.

Among transcription factors that bind to IFN-stimulated response elements and regulate IFN-induced expression of IFITs, the STAT1, STAT2, and IFN regulatory factors-9 play crucial roles [10]. In addition to these molecules, IRF-1 can induce IFIT3 gene expression through either IRF-9/STAT2-dependent or IRF-9/STAT2-independent mechanisms [30]. In the present study, we showed that knockdown of STAT2 but not STAT1 or STAT3 reduced DV-induced IFIT3 expression. In addition to being important downstream signaling molecules of IFNs, STAT proteins are targeted and downregulated by DV to evade immunosurveillance [20,31,32]. Here, we provided another example demonstrating that similar to STAT proteins, DV could also regulate IFN-α-induced IFIT3 expression. Under similar conditions, DV did not affect IFN-γ-induced IFIT3 expression. Currently, the significance of downregulation of IFN-α-induced IFIT3 expression by DV is not exactly clear.

Our results also support previous observations concerning the anti-viral effects of IFIT3 in vesicular stomatitis virus and murine encephalomyelitis virus infections [33]. IFIT3 has been shown to interact with other IFIT family members and several related signaling molecules such as TBK1 and IRF3, and thereby further amplify signaling events [34,35]. For example, in HCV infection, synergies between IFIT1 and IFIT3 were demonstrated to specifically inhibit viral entry or intracellular trafficking, and attenuate viral replication [36]. In addition, a complex formed by the combination of IFIT1, IFIT2, and IFIT3 can recognize 5'-triphosphate RNA, which is a microbial structure recognized by antiviral innate immunity [34]. The binding of viral RNA by IFIT1 further suggests the possibility that this effect may be shared by all IFITs, because they all contain nucleotide binding regions [34]. In agreement with these observations, overexpression of IFIT3 reduced viral production. This suggests that higher amounts of IFIT3 produced additional anti-viral effects in the example of DV infection.

It is intriguing to observe that knockdown of IFIT3 caused cell death and yet enhanced viral production in DV-infected cells. As a common sense, it is usually hard for viruses to propagate in dying cells. However, the data are quite consistent and with statistical significance. There are few explanations. First, we do not consider that DV can replicate efficiently in dying cells but rather viral replication or increased viral load causes cell death. In deficiency of anti-viral effects (not necessary from IFN-α) in IFIT3 knockdown cells, virus propagated more efficiently and thus led to increased virus load. Second, knockdown of IFIT3 had a tendency causing reduced production of anti-viral cytokine like IFN-β although the statistical analysis failed to show significance (Figure S3C). This added another factor to increase viral production. Third, the experiments examining the kinetics of viral production and cell death with or without deficiency of IFIT3 revealed that viral production peaked as early as 24 h after infection and the prominent effect of IFIT3 knockdown causing cell death appeared late at around 48-72 h after virus infection (Figure S3B). The net results from the interplay of these factors may come up with increased viral load. How to adjust the weight of these factors on net viral production is currently difficult to answer. Alternatively, in addition to the factors mentioned above, knockdown of IFIT3 may also result in inhibition of IFIT3 downstream signaling pathways and part of these pathways may be responsible for inhibition of viral replication. We are currently investigating how many possible anti-viral pathways are involved in DV-infected cells with deficiency of IFIT3.

In conclusion, in the present study, we demonstrated that IFIT3 induced after IFN stimulation may be critical for maintaining cell survival, and that a deficiency of this molecule resulted in increased rates of apoptotic cell death, which were exaggerated by DV infection. We further demonstrated the crucial role of STAT2 in regulating DV-induced IFIT3 expression. Moreover, similar to STAT proteins targeted by DV to suppress the anti-viral effects of IFN-α, IFIT3 was also targeted by DV; however the subsequent effects and mechanisms might be different. Although DV might regulate IFIT3 expression to modulate IFN-α-mediated immune response, IFIT3 is not absolutely required for anti-viral replication by IFN-α. Thus, the biological significance of downregulation of IFN-α-induced IFIT3 by DV infection is currently not clear. Collectively, our study contributes new insights for understanding the functions and roles of IFIT3 - a member of the *ISG* family. 

## Supporting Information

Figure S1 **Blockade of DV-induced IFIT3 induction by neutralizing monoclonal antibodies recognizing IFN receptor.** A549 cells at 1х10^5^ cells/mL were pretreated with MMHAR2 (IFN receptor neutralizing antibody, 3 μg/ mL) or control antibody for 2 h and then infected by mock or DV at M.O.I. = 5 or treated with 100 units/mL IFN-α for additional 24 h. The total cell lysates were collected and the expression of IFIT3 or β-actin was determined by western blotting (A). The relative band intensity was measured and shown in (B) and (C). Data show representative results and analyses pooled from 5 independent experiments. The analysis was performed by ANOVA as described in Materials and Methods. *P < 0.05, **P < 0.01, ***P < 0.001.
(TIFF)

Figure S2 **Induction of IFIT3 is STAT-2-dependent.** A549 cells were infected by mock or DV for 3, 6, and 24 h and protein levels of both phosphorylated and non-phosphorylated STAT1, STAT2, and STAT3 were analyzed by western blotting and the band intensity was calculated and shown (A). Expression of IFIT3 in DV-infected A549 cells with knockdown of either STAT2 (B), STAT1 (C) or STAT3 (D) was determined by western blotting and the band intensity was calculated and shown. Data show the analyses pooled from at least 3 independent experiments. The analysis was performed by ANOVA as described in Materials and Methods. *P < 0.05, ***P < 0.001. n.s: no significance. Ctl stands for control.
(TIFF)

Figure S3 **Effects of IFIT3 knockdown on virus production and cell death.** A549 cells were infected by DV at M.O.I. = 5 (A) or transfected with control siRNA (si-Ctl) or IFIT3 siRNA (siIFIT3-2) (B) for 16, 24, 48 or 72 h. The cells were collected for determining cell death by sub-G1 analysis or the supernatants for determining virus titers by plaque assays. In (C), A549 cells were transfected with IFIT3 siRNA for 24 h and then infected by mock or DV at M.O.I. = 0.5 or 5 for additional 13 h. The expression of mRNA of IFN-β was determined by quantitative RT/PCR. The representative results and the analysis pooled from at least three independent experiments were shown. Ctl stands for control.
(TIFF)

Figure S4 **The induction of apoptotic molecules by deficiency of IFIT3.** A549 cells transfected with control siRNA (si-Ctl) or IFIT3 siRNA (siIFIT3-2) for 24 h were infected by mock or DV at M.O.I. = 5 for another 24 h. The cleaved proteins, including caspase 8, caspase 9, caspase 3 and BAX were determined by western blotting shown in Figure 5A. The relative band intensities of cleaved proteins were quantified (A). The synergistic effects between DV infection and the IFIT3 knock-down were calculated and shown in (B). The representative results and the analysis pooled from at least three independent experiments were shown. The analysis was performed by ANOVA as described in Materials and Methods. *P<0.05, **P<0.01, ***P<0.001. Ctl stands for control.
(TIFF)

Figure S5 **DV infection within 6 h had a tendency to gain ability to downregulate IFN-α-induced IFIT3 expression.** DV-infected A549 cells (1 x 10^5^/mL) were treated with 1000 units IFN-α at 6 h after virus infection and incubated for additional 18 h. The expression of IFIT3 was determined by western blotting. The right panel showed the relative band intensity of IFIT3. Data show representative results and analyses pooled from 3 independent experiments. The analysis was performed by ANOVA as described in Materials and Methods. n.s: no significance.
(TIFF)

Figure S6 **The knockdown of IFIT3 did not reduce the potency of the anti-viral protection of IFN-α.** A549 cells transfected with control siRNA (si-Ctl) or IFIT3 siRNA (siIFIT3-2) for 24 h were pretreated with 100 units/mL (A and B) or 1000 units/mL (B) of IFN-α for 5 h and then infected by mock or DV at M.O.I.= 0.5 (B) or 5 (A and B) for another 24 or 48 h. The supernatants were collected for determining virus titers by plaque assays (A). The cells were collected at 48 h postinfection for the measurement of expression of intracellular NS3 by flow cytometry (B). The representative result and the analysis pooled from at least three independent experiments are shown. The analysis was performed by ANOVA as described in Materials and Methods. *P<0.05, **P<0.01, ***P<0.001. Ctl stands for control.
(TIFF)

Figure S7 **A cartoon shows how IFIT3 regulated DV production and cell death in A549 cells.** DV infection induced production of IFNs from A549 cells. The binding of IFN to the receptor induced mRNA expression and protein production of IFIT3 through a STAT2-dependent mechanism. The deficiency of IFIT3 enhanced DV-induced apoptotic cell death by inducing cleavage of pro-apoptotic molecules such as BAX, caspase 3, 8 and 9. The deficiency of IFIT3 also increased viral production in A549 cells. Overexpression of IFIT3 by itself modestly reduced viral replication.
(TIFF)
