# Supplementary figures and images for: Protective Roles of Interferon-Induced Protein with Tetratricopeptide Repeats 3 (IFIT3) in Dengue Virus Infection of Human Lung Epithelial Cells
Source: PLoS One. 2013 Nov 4;8(11):e79518. doi: 10.1371/journal.pone.0079518 (PMC3817122; doi:10.1371/journal.pone.0079518)

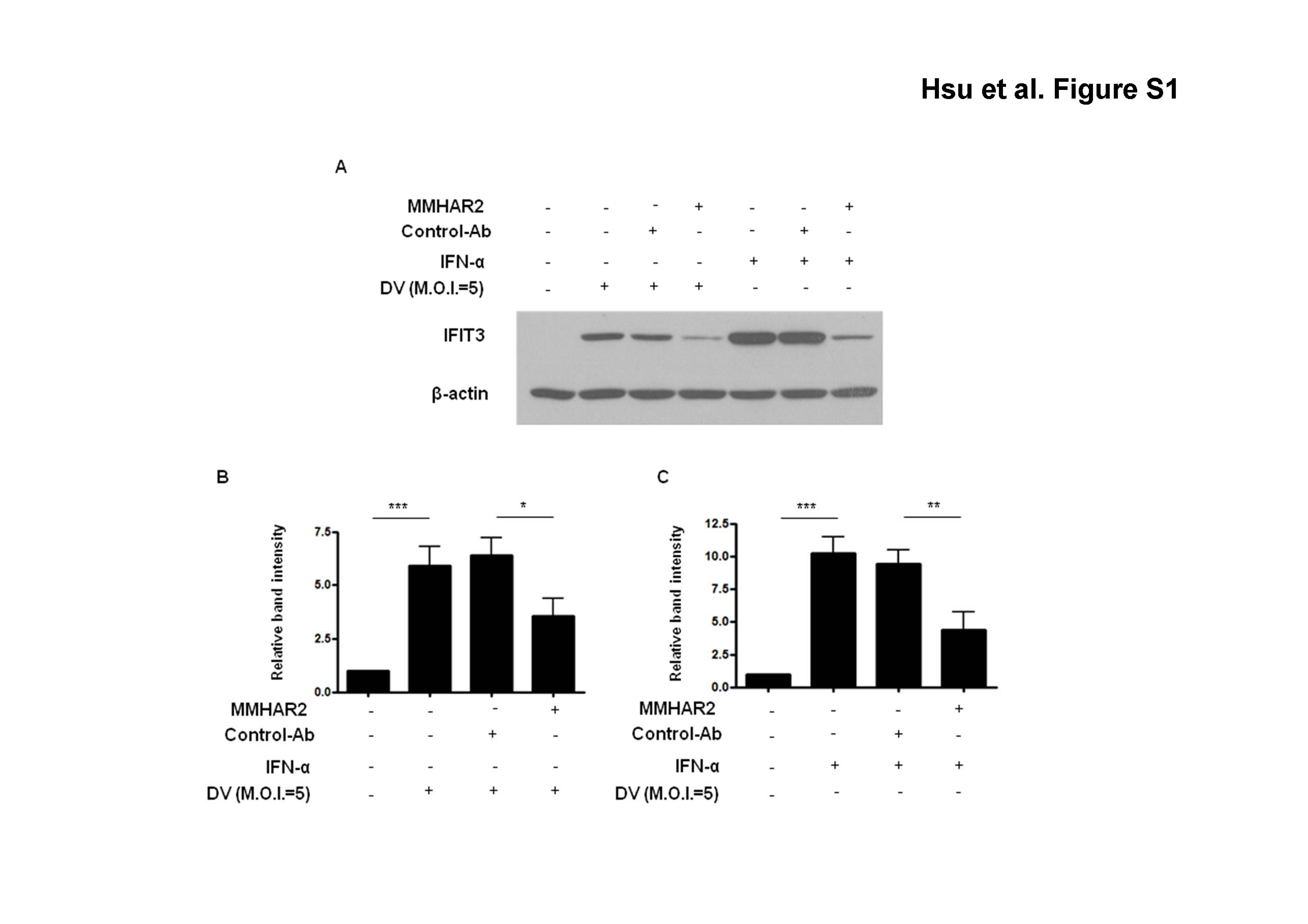

Supplement: Figure S1 — Blockade of DV-induced IFIT3 induction by neutralizing monoclonal antibodies recognizing IFN receptor. A549 cells at 1х105 cells/mL were pretreated with MMHAR2 (IFN receptor neutralizing antibody, 3 μg/ mL) or control antibody for 2 h and then infected by mock or DV at M.O.I. = 5 or treated with 100 units/mL IFN-α for additional 24 h. The total cell lysates were collected and the expression of IFIT3 or β-actin was determined by western blotting (A). The relative band intensity was measured and shown in (B) and (C). Data show representative results and analyses pooled from 5 independent experiments. The analysis was performed by ANOVA as described in Materials and Methods. *P < 0.05, **P < 0.01, ***P < 0.001. (TIFF) [file pone.0079518.s001.tiff]

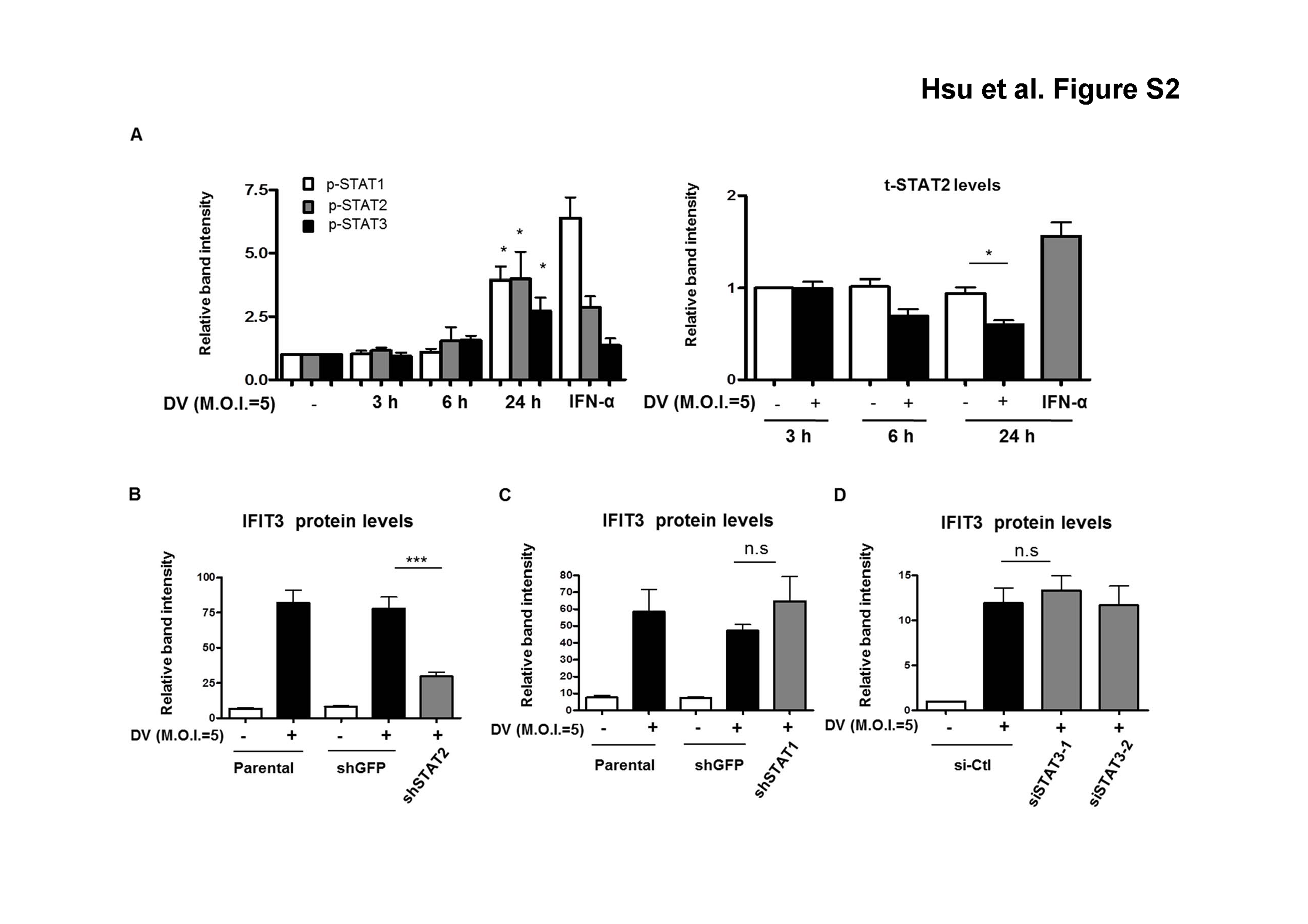

Supplement: Figure S2 — Induction of IFIT3 is STAT-2-dependent. A549 cells were infected by mock or DV for 3, 6, and 24 h and protein levels of both phosphorylated and non-phosphorylated STAT1, STAT2, and STAT3 were analyzed by western blotting and the band intensity was calculated and shown (A). Expression of IFIT3 in DV-infected A549 cells with knockdown of either STAT2 (B), STAT1 (C) or STAT3 (D) was determined by western blotting and the band intensity was calculated and shown. Data show the analyses pooled from at least 3 independent experiments. The analysis was performed by ANOVA as described in Materials and Methods. *P < 0.05, ***P < 0.001. n.s: no significance. Ctl stands for control. (TIFF) [file pone.0079518.s002.tiff]

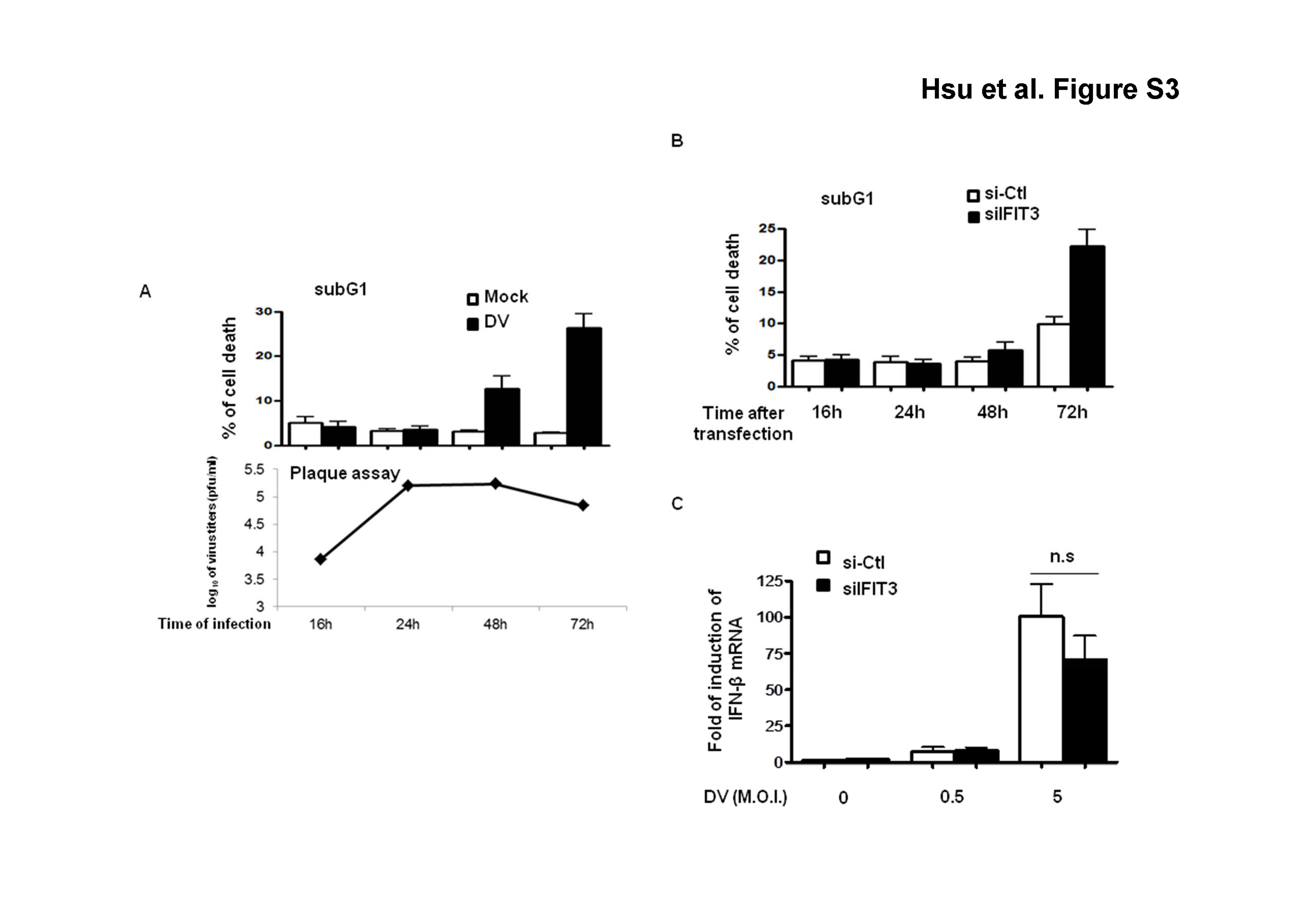

Supplement: Figure S3 — Effects of IFIT3 knockdown on virus production and cell death. A549 cells were infected by DV at M.O.I. = 5 (A) or transfected with control siRNA (si-Ctl) or IFIT3 siRNA (siIFIT3-2) (B) for 16, 24, 48 or 72 h. The cells were collected for determining cell death by sub-G1 analysis or the supernatants for determining virus titers by plaque assays. In (C), A549 cells were transfected with IFIT3 siRNA for 24 h and then infected by mock or DV at M.O.I. = 0.5 or 5 for additional 13 h. The expression of mRNA of IFN-β was determined by quantitative RT/PCR. The representative results and the analysis pooled from at least three independent experiments were shown. Ctl stands for control. (TIFF) [file pone.0079518.s003.tiff]

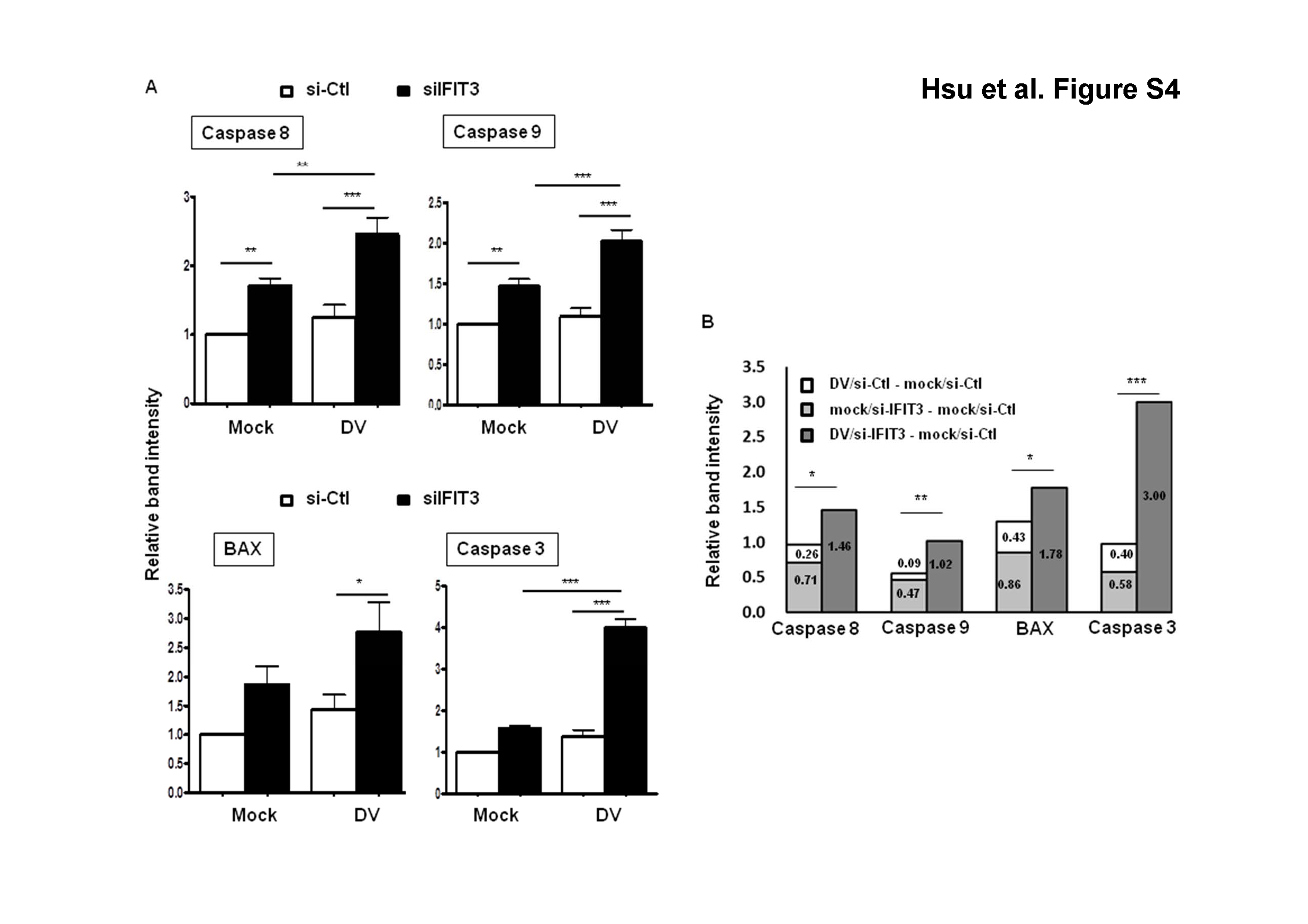

Supplement: Figure S4 — The induction of apoptotic molecules by deficiency of IFIT3. A549 cells transfected with control siRNA (si-Ctl) or IFIT3 siRNA (siIFIT3-2) for 24 h were infected by mock or DV at M.O.I. = 5 for another 24 h. The cleaved proteins, including caspase 8, caspase 9, caspase 3 and BAX were determined by western blotting shown in Figure 5A. The relative band intensities of cleaved proteins were quantified (A). The synergistic effects between DV infection and the IFIT3 knock-down were calculated and shown in (B). The representative results and the analysis pooled from at least three independent experiments were shown. The analysis was performed by ANOVA as described in Materials and Methods. *P<0.05, **P<0.01, ***P<0.001. Ctl stands for control. (TIFF) [file pone.0079518.s004.tiff]

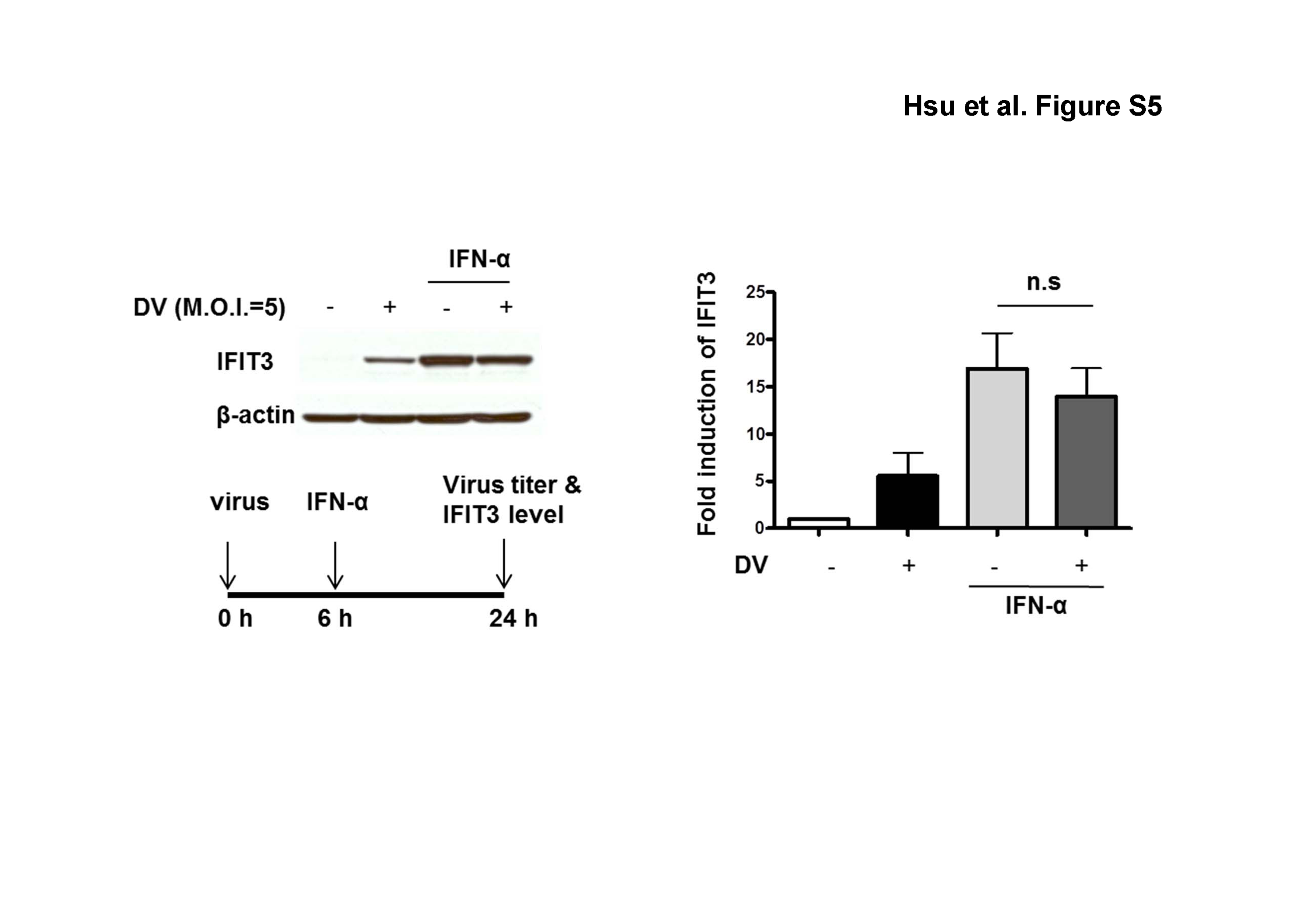

Supplement: Figure S5 — DV infection within 6 h had a tendency to gain ability to downregulate IFN-α-induced IFIT3 expression. DV-infected A549 cells (1 x 10^5^/mL) were treated with 1000 units IFN-α at 6 h after virus infection and incubated for additional 18 h. The expression of IFIT3 was determined by western blotting. The right panel showed the relative band intensity of IFIT3. Data show representative results and analyses pooled from 3 independent experiments. The analysis was performed by ANOVA as described in Materials and Methods. n.s: no significance. (TIFF) [file pone.0079518.s005.tiff]

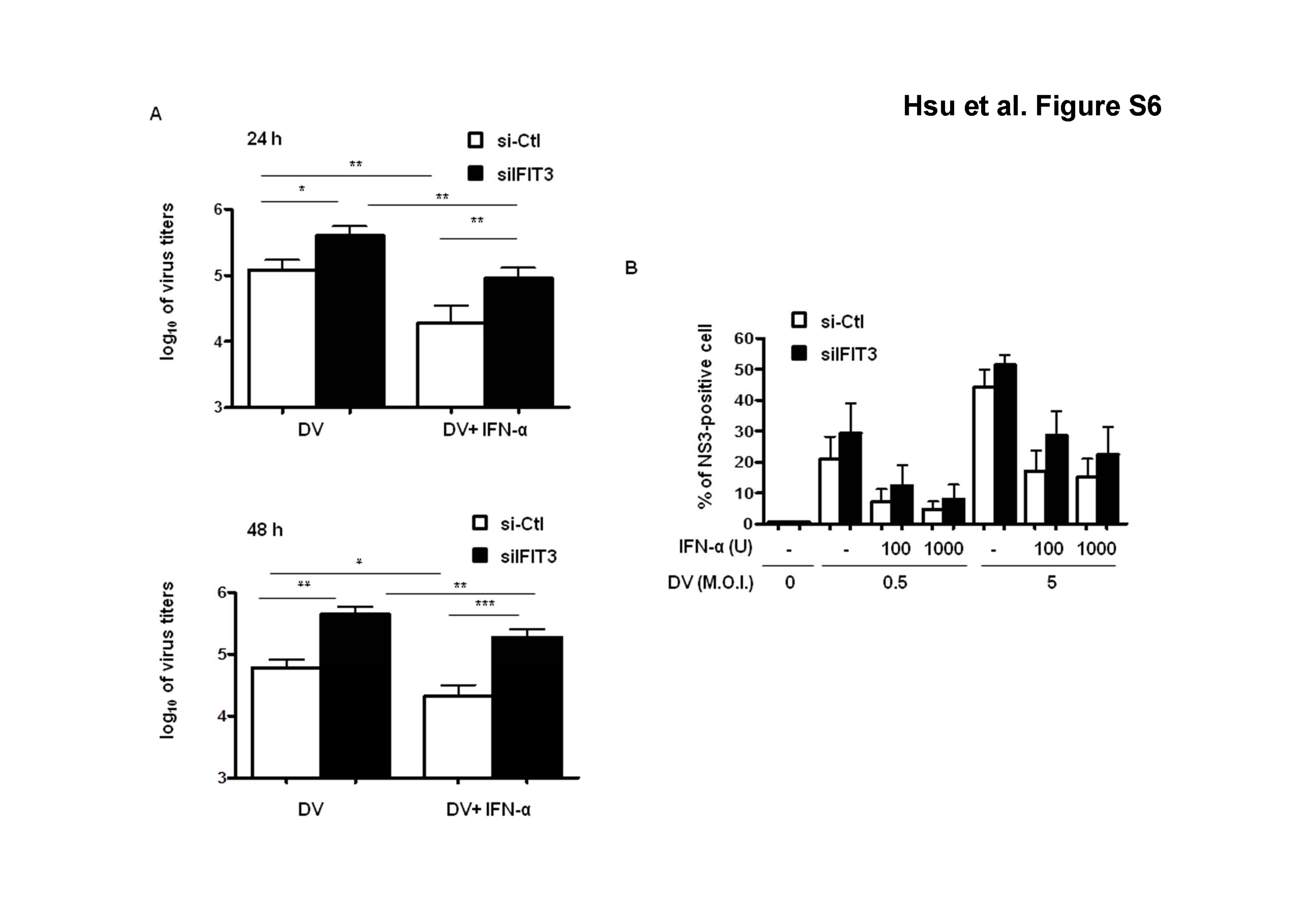

Supplement: Figure S6 — The knockdown of IFIT3 did not reduce the potency of the anti-viral protection of IFN-α. A549 cells transfected with control siRNA (si-Ctl) or IFIT3 siRNA (siIFIT3-2) for 24 h were pretreated with 100 units/mL (A and B) or 1000 units/mL (B) of IFN-α for 5 h and then infected by mock or DV at M.O.I.= 0.5 (B) or 5 (A and B) for another 24 or 48 h. The supernatants were collected for determining virus titers by plaque assays (A). The cells were collected at 48 h postinfection for the measurement of expression of intracellular NS3 by flow cytometry (B). The representative result and the analysis pooled from at least three independent experiments are shown. The analysis was performed by ANOVA as described in Materials and Methods. *P<0.05, **P<0.01, ***P<0.001. Ctl stands for control. (TIFF) [file pone.0079518.s006.tiff]

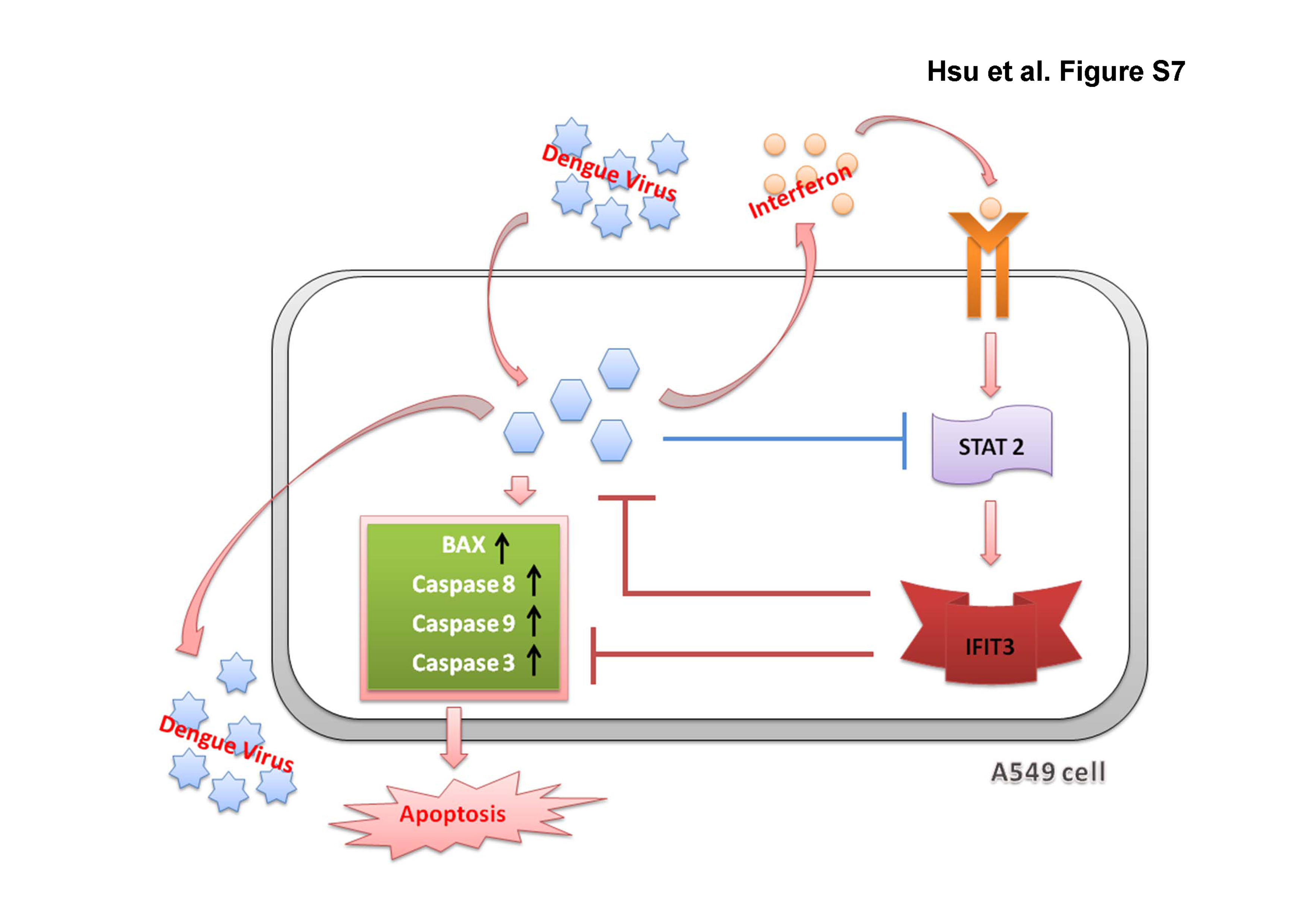

Supplement: Figure S7 — A cartoon shows how IFIT3 regulated DV production and cell death in A549 cells. DV infection induced production of IFNs from A549 cells. The binding of IFN to the receptor induced mRNA expression and protein production of IFIT3 through a STAT2-dependent mechanism. The deficiency of IFIT3 enhanced DV-induced apoptotic cell death by inducing cleavage of pro-apoptotic molecules such as BAX, caspase 3, 8 and 9. The deficiency of IFIT3 also increased viral production in A549 cells. Overexpression of IFIT3 by itself modestly reduced viral replication. (TIFF) [file pone.0079518.s007.tiff]
